# Beyond prediction: unveiling the prognostic power of μ-opioid and cannabinoid receptors, alongside immune mediators, in assessing the severity of SARS-CoV-2 infection

**DOI:** 10.1186/s12879-024-09280-6

**Published:** 2024-04-12

**Authors:** Masoumeh Tavakoli-Yaraki, Aida Abbasi, Fatemeh Nejat Pishkenari, Saeed Baranipour, Alireza Jahangirifard, Seyed Bashir Mirtajani, Zahra Noorani Mejareh, Mohammad Amin Vaezi, Jila Yavarian, Bahare Abdollahi, Talat Mokhtari-Azad, Vahid Salimi

**Affiliations:** 1 Department of Biochemistry, School of Medicine, Iran University of Medical Sciences, Tehran, Iran; 2 Department of Virology, School of Public Health, Tehran University of Medical Sciences, Tehran, P.O. Box: 1417613151, Iran; 3 Lung Transplant Research Center, National Research Institute of Tuberculosis and Lung Diseases (NRITLD), Shahid Beheshti University of Medical Sciences, Tehran, Iran; 4 Student Research Committee, School of Medicine, Iran University of Medical Sciences, Tehran, Iran

**Keywords:** Cannabinoid receptor, Opioid receptor, MCP-1, IL-17, IFN-γ, Osteopontin, SARS-CoV-2, Predictor

## Abstract

**Background:**

This study aims to explore the potential of utilizing the expression levels of cannabinoid receptor 2 (CB2), μ-opioid receptor (MOR), MCP-1, IL-17, IFN-γ, and osteopontin as predictors for the severity of SARS-CoV-2 infection. The overarching goal is to delineate the pathogenic mechanisms associated with SARS-CoV-2.

**Methods:**

Using quantitative Real-time PCR, we analyzed the gene expression levels of CB2 and MOR in nasopharynx specimens obtained from patients diagnosed with SARS-CoV-2 infection, with 46 individuals classified as having severe symptoms and 46 as non-severe. Additionally, we measured the circulating levels of MCP-1, IL-17, IFN-γ, and osteopontin using an ELISA assay. We examined the predictive capabilities of these variables and explored their correlations across all patient groups.

**Results:**

Our results demonstrated a significant increase in MOR gene expression in the epithelium of patients with severe infection. The expression of CB2 receptor was also elevated in both male and female patients with severe symptoms. Furthermore, we observed concurrent rises in MCP-1, IL-17, IFN-γ, and osteopontin levels in patients, which were linked to disease severity. CB2, MOR, MCP-1, IL-17, IFN-γ, and osteopontin showed strong predictive abilities in distinguishing between patients with varying degrees of SARS-CoV-2 severity. Moreover, we identified a significant correlation between CB2 expression and the levels of MOR, MCP-1, osteopontin, and IFN-γ.

**Conclusions:**

These results underline the interconnected nature of molecular mediators in a sequential manner, suggesting that their overexpression may play a role in the development of SARS-CoV-2 infections.

**Graphical Abstract:**

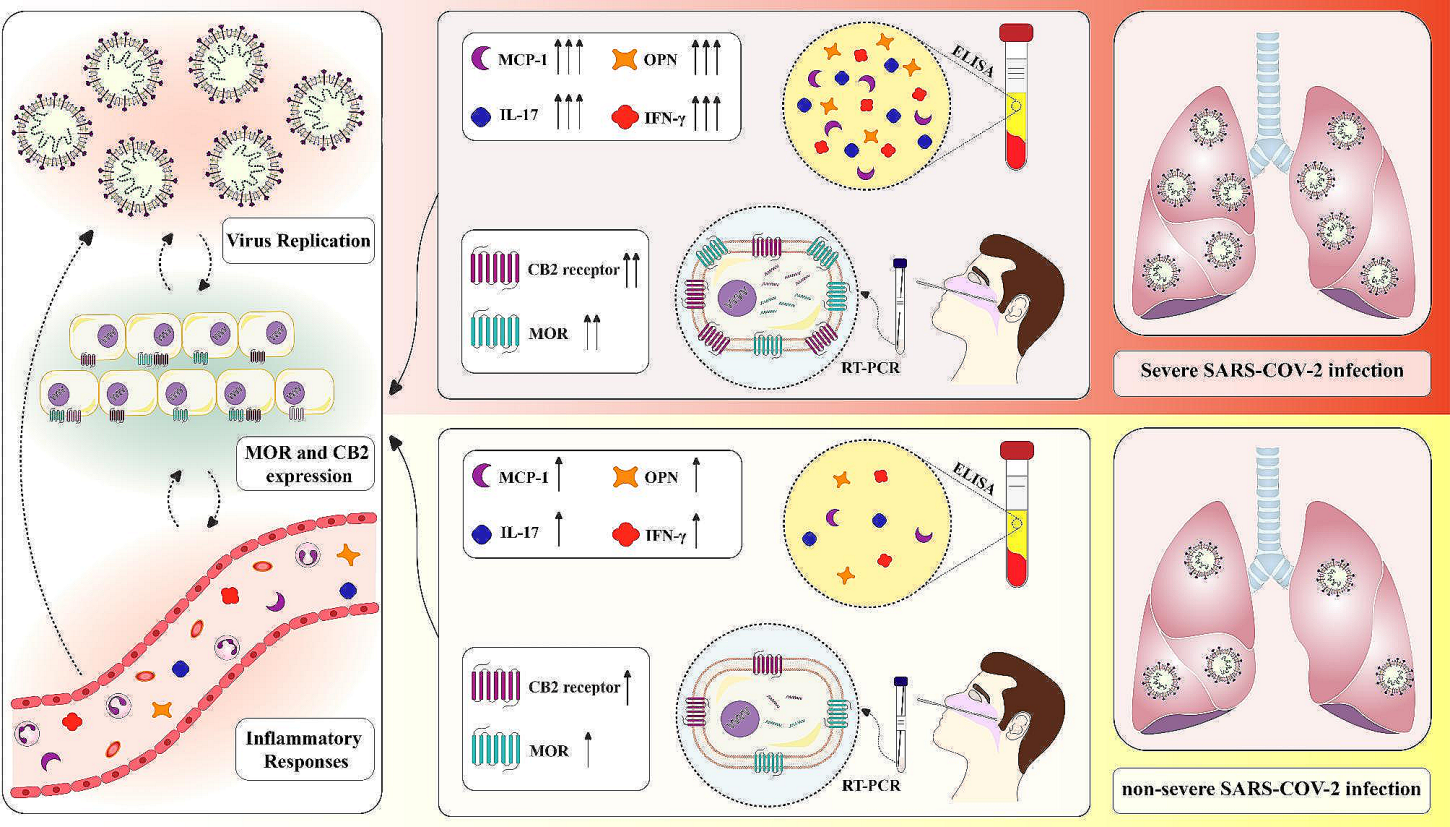

## Introduction

The global impact of the severe acute respiratory syndrome coronavirus 2 (SARS-CoV-2) infection has led to substantial public health challenges, with elevated rates of illness and death worldwide [1]. Ongoing research efforts to comprehend the pathogenesis of COVID-19 highlight the critical role of the host immune response in determining infection severity and disease outcomes [2, 3].

Recent studies have underscored the significance of the opioid and cannabinoid systems in regulating immune system responses, influencing factors such as inflammatory cytokine production [4], cell growth regulation [5, 6], and immune system balance [7]. The cannabinoid receptors, specifically CB1 and CB2, belonging to the G-protein coupled receptor (GPCR) family, have distinct distribution patterns and biological functions [8]..

CB2, in particular, plays a pivotal role in immune response by mitigating inflammation and modulating signaling pathways [7, 9].. Similarly, the μ-opioid receptor (MOR), another GPCR member, exerts various effects on the immune and nervous systems, as well as cell growth regulation [10–12].

In the context of viral infections, inflammation plays a crucial role, and targeting these receptors could offer therapeutic benefits in managing viral diseases [13, 14]. Research has shown that blocking the CB2 receptor during respiratory syncytial virus (RSV) infection led to altered immune cell responses and increased damage in the lungs, indicating a potential link between CB2 and RSV pathology. Conversely, activation of CB receptors during influenza infection led to reduced immune cell recruitment but increased viral replication, hinting at their involvement in viral replication processes [15]..

Additionally, studies in Balb/c mice infected with RSV post-vaccination demonstrated that blocking the opioid receptor resulted in heightened immune responses and exacerbated infection outcomes, while also enhancing vaccine efficacy in reducing reinfection rates [16].. Blocking the opioid receptor with nalmefene in Balb/c mice infected with respiratory syncytial virus (RSV) post-vaccination (FI-RSV) led to elevated IFN-gamma levels, increased immune cell presence in BAL fluid, and intensified host immune response. Despite this, the blockade enhanced the vaccine’s effectiveness in lowering reinfection rates. These findings underscore the complex interplay between opioid and cannabinoid receptors in immune modulation and viral pathogenesis [10, 15, 17].. In this context, the opioid/cannabinoid systems have gained recognition for their immunomodulatory effects and their regulatory functions in cell growth, apoptosis, as well as the modulation of inflammatory cytokines and regulatory T cells [9, 10, 18, 19]. It is important to highlight that the expression of CB receptors can be triggered by innate immune cells, leading to the production of endogenous cannabinoids and the regulation of homeostasis and inflammatory responses [20]. Additionally, opioids have been observed to exhibit both immunosuppressive and immune stimulatory effects, which can vary depending on the specific type of opioids, opioid receptors, host factors, dosage, and timing of administration [21]. It should be noted that inflammatory and immune responses can have diverse effects on viral replication, either compromising and eradicating it or facilitating it, depending on the type of virus and infection [7, 19].

Despite attempts made, there remains a gap in understanding the potential influence of these receptors on the progression and intensity of SARS-CoV-2. It is crucial to comprehend the involvement of opioid and cannabinoid receptors in COVID-19 infection to enhance clinical care and advance treatment strategies. Various biomarkers were tested as predictors for the severity of COVID-19 [22–24]; however, little is known about utilizing MOR and CB2 receptors as predictors of severity.

Hence, this study aims to examine the expression of MOR and CB2 receptors, along with the levels of associated cytokines and modulators, in both pharyngeal epithelial cells and blood samples from patients with varying degrees of COVID-19 infection severity. The objective is to assess the potential predictive value of these factors and their correlations with the severity of SARS-CoV-2 infection.

## Materials & methods

### Patients features and sample collection

Ninety-two participants were included in this study, with 46 experiencing severe SARS-CoV-2 infection and the remaining 46 having non-severe SARS-CoV-2 infection. Sample gathering, analysis, and variable evaluation were carried out following the guidelines of the Declaration of Helsinki, our institution’s ethical approval [25],, and in alignment with our prior research [26]. The research focused on adult individuals aged 18 and above who exhibited COVID-19 respiratory symptoms and tested positive for SARS-CoV-2 through PCR testing. The patient enrollment took place from September 2020 to October 2022 from National Research center of Tuberculosis and Lung Diseases. Nasopharynx specimens were collected from each patient, and viral RNA was extracted using the High Pure Viral RNA Kit (Roche Life Sciences). The virus presence was confirmed through RT-PCR testing of SARS-CoV-2 nucleoprotein (N) and ORF1ab genes, following national and established assessing protocols. The study excluded patients with clinical complications like immune system deficiencies, concurrent viral or bacterial infections, pregnancy, and breastfeeding. Patients were divided into two groups based on clinical symptoms, radiographic and laboratory results, and the viral load in nasopharyngeal samples. The severe group included patients with acute symptoms, high virus concentration (CT value < 20), and required hospitalization. The non-severe group had mild symptoms, low virus concentration (CT value > 30), and did not require hospitalization. Notably, all patients were evaluated at their first visit to clinics, and the samples were collected before hospitalization and none of the patients had received COVID-19 vaccine at the time of investigation and sampling. The study focused on patients who were not currently undergoing prescribed or self-administered drug therapy and were in a fasting state during sampling. Table 1 outlines the demographic characteristics of these patients, detailing their age, gender, and clinical symptoms. The gender distribution was equal, with 78.26% of both male and female patients being above 40 years old. Male patients with severe infection had a mean age of 47.78 ± 11.83, while female patients had a mean age of 49.55 ± 15.67. Patients with a higher degree of disease severity displayed more intense clinical symptoms, including fever and muscle pain, which were present in approximately 70% of cases. Furthermore, more than 70% of these individuals experienced symptoms like cough, sore throat, and fatigue; in contrast, symptoms like vomiting and diarrhea were less commonly reported among this group of patients.

**Table 1:** Demographic features of the enrolled patients

Variable	Groups	Severe patients (*N* = 46)	Non-Severe patients (*N* = 46)	
Gender (N,%)	Male Female	23(50%) 23(50%)	23(50%) 23(50%)	
Age (N, %)	≤40 40<	10(21.7%) 36(78.26%)	10(21.7%) 22(78.26%)	
Age (Mean ± SD)		47.78 ± 11.83	49.55 ± 15.67	
Weight (Mean ± SD)	Male Female	73.32 ± 10.32 70 ± 11.31	74.21 ± 11.65 71.42 ± 11.12	
Fever	Yes No	32(69.56%) 14(30.44%)	27(58.69%) 19(41.31%)	
Dyspnea	Yes No	26(56.52%) 20(43.48%)	24(52.17%) 22(47.83%)	
Cough	Yes No	36(78.26%) 10(21.7%)	25(54.34%) 24(45.46%)	
Sore throat	Yes No	33(71.73%) 13(28.28%)	27(58.69%) 19(41.31%)	
Myalgia	Yes No	31(67.39%) 15(32.61%)	21(45.65%) 25(54.35%)	
Headache	Yes No	20(43.48%) 26(56.52%)	17(36.95%) 29(63.04%)	
Tiredness	Yes No	34(73.91%) 12(26.09%)	24(52.17%) 22(47.83%)	
Diarrhea	Yes No	11(23.91%) 35(76.09%)	2(4.34%) 44(95.65%)	
Vomiting	Yes No	9(19.56%) 37(80.43%)	4(8.69%) 42(91.30%)	

** The table presents demographic features of the enrolled patients categorized as severe (*N* = 46) and non-severe (*N* = 46) for SARS-CoV-2 infection. Gender distribution, age groups, mean age, and the presence of symptoms such as fever, dyspnea, cough, sore throat, myalgia, headache, tiredness, diarrhea, and vomiting are outlined

Gender distribution shows an equal split between male and female patients in both severe and non-severe groups

Age distribution indicates a higher proportion of patients aged 40 and above in both severity categories

Fever, cough, sore throat, myalgia, and tiredness were common symptoms reported by patients, with varying prevalence between severe and non-severe cases

Dyspnea, headache, diarrhea, and vomiting were also noted, with differences in occurrence between severe and non-severe groups

Mean age was slightly higher in the non-severe group compared to the severe group

These demographic features offer insights into the clinical profile of patients enrolled in the study, highlighting key characteristics relevant to disease severity assessment

Analysis methods for each entry in the table include statistical tests for group comparisons and descriptive statistics for demographic variables

### CB2 receptor and MOR gene expression analysis

To measure the gene expression level of CB2 and MOR, the total RNA of the epithelial cells of patients was extracted from the nasopharyngeal swab specimens using TRIzol (Invitrogen). The cDNA Synthesis Kit (Roche Life Sciences) and the SYBR Premix Ex Taq II (Takara, Japan) were applied for cDNA synthesis and gene expression analysis, respectively. The details of methodology, primer sequences and analysis are described in our previously published studies [26–28].

### Assessment the serum levels of interleukin-17, MCP-1, osteopontin and IFN-γ

Patients’ serum levels of IL-17, MCP-1, OPN, and IFN-γ were assessed using the ELISA technique, in accordance with the manufacturer’s instructions. A sandwich ELISA technique was employed for all variables, utilizing a conjugate of Streptavidin-horseradish peroxidase (HRP), specific capture antibodies, and detection antibodies. Samples and standards, each at a volume of 100μL, were simultaneously introduced into assigned wells on the plate and left to incubate for 2 h. Post incubation, the wells were carefully aspirated and washed before the addition of 100μL of detection antibody to each well, followed by another 2-hour incubation period. After thorough washing, 100μL of a streptavidin-HRP solution was added to each sample and incubated for 20 min in a light-protected environment. Subsequently, a substrate solution of 100μL was introduced, and after an additional washing step, the reaction in each well was halted by the addition of 50 μL of stop solution. The optical density at 450 nm was then determined using a microplate reader. A human IL-17 RA/IL-17 R quantitative ELISA kit (R&D# DY177), an Osteopontin (OPN) quantitative kit (R&D# DOST00), IFN-γ quantitative kit (R&D# DIF50C) and CCL2/MCP-1 quantitative kit (R&D# DY279-05) were applied for detection with an analytical sensitivity of 31.2 pg/mL, 0.3 ng/mL, 5.69 pg/mL and15.6 pg/mL for lL-17, OPN, IFN-γ and MCP-1, respectively.

### Statistical analysis

Statistical analysis was conducted using Graph Pad Prism Version 6 (Graph Pad Software, San Diego, California). Continuous data were reported as median (range), while discrete variables were presented as numbers (%). The Kolmogorov-Smirnov test was selected for assessing normality due to its robustness and suitability for larger sample sizes. The Kolmogorov-Smirnov test is known for its effectiveness in detecting departures from normality across various distributions, making it a reliable choice for our dataset.

Additionally, before employing non-parametric tests, data transformations were explored to ensure the validity of the statistical analysis. The nonparametric Mann-Whitney U-test was employed to analyze differences in gene expressions, and the parametric unpaired t-test was used to compare the levels of IL-17, MCP-1, OPN, and IFN-γ between groups. Correlation analysis was performed using Pearson analysis if both variables had a normal distribution, and Spearman analysis was utilized if one or both variables had a non-normal distribution. The ability to differentiate markers between the investigated groups were analyses using receiver operating characteristic (ROC) curve analysis, and the area under the curve (AUC) and optimal cut-off values were determined based on the Youden index [29]. Separate ROC analyses were conducted for each marker to assess the discriminative power between male patients with varying infection intensities and to evaluate the differentiation capability between female patients with different infection intensities for each marker individually. The ROC analysis was performed using Statistical Package for Social Science software (SPSS v.16) for statistical analysis and *P*-values < 0.05 (two-tailed) were considered statistically significant.

Binary multivariate logistic regression was utilized to examine the predictive values of the variables in disease severity. The regression coefficient (B) and odds ratio (OR) were employed to gauge the degree of association of biomarkers with mortality in the regression model. Statistical significance was determined by *P*-values < 0.05 (two-tailed).

## Results

### The CB2 receptor and MOR expression levels in patients

**Fig. 1:**
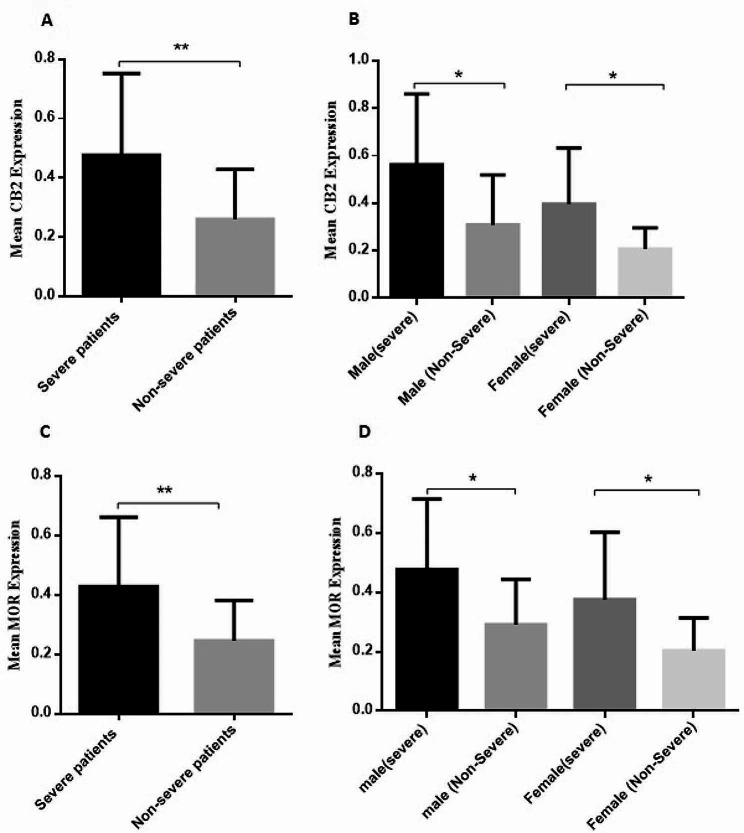
CB2 receptor and MOR expression pattern in patients Relative expression of Cannabinoid receptor 2 (CB2 receptor) and MOR (μ-opioid receptor) was evaluated in patients with different disease severity using specific CB2 and MOR primers by real-time PCR and normalized to the level of β-actin, as a housekeeping gene. Severe patient had acute clinical manifestations of the disease and high virus concentration in the nasopharyngeal specimen (CT value of gene expression below 20) and were hospitalized; while, non-severe patients had mild respiratory symptoms and low virus concentration in the nasopharyngeal specimen (CT value of gene expression greater than 30) and were not assessed for hospitalization. (**A**): An elevated level of CB2 receptor was detected in severe patients; (**B**): The CB2 receptor expression level increased in male and female patients with severe infection versus their opposite groups. (**C**): The mRNA level of MOR enhanced significantly in patients with severe infection; (**D**): MOR gene expression enhanced in male and female patients with severe SARS-CoV-2 (Severe acute respiratory syndrome coronavirus 2) infection versus their non-severe counterparts. Statistical significance was determined by *P*-values (two-tailed) and showed by asterisks on bars (*= *P* < 0.05, **= *P* < 0.01)

Among patients diagnosed with SARS-CoV-2, individuals with severe infection exhibited notably elevated levels of CB2 receptors in nasopharyngeal swabs compared to those with milder symptoms (*P* = 0.0019) (Fig. 1.A). In patients with severe infection, the average mRNA level of CB2 receptors was 0.47 ± 0.05, while in patients with non-severe infection, it was 0.26 ± 0.03. In male patients, those with severe infection exhibited a notably higher mRNA level of CB2 receptors (0.56 ± 0.08) compared to male patients with non-severe infection (0.30 ± 0.06) (*P* = 0.02) (Fig. 1.B). Likewise, female patients with severe infection (0.3 ± 0.06) demonstrated elevated CB2 receptor levels compared to female patients with non-severe infection (0.20 ± 0.02) (*P* = 0.01) (Fig. 1.B).Furthermore, the expression level of MOR was evaluated in patients with different SARS-CoV-2 severity, revealing that the mRNA level of MOR was higher in patients with severe infection (0.42 ± 0.04) compared to non-severe infection (0.24 ± 0.02) (*P* = 0.002) (Fig. 1.C). Additionally, male patients with severe infection demonstrated a significantly elevated mRNA level of MOR (0.47 ± 0.06) compared to their non-severe counterparts (0.29 ± 0.02) (*P* = 0.03) (Fig. 1.D). A similar pattern of expression was observed in female patients, with severe patients (0.37 ± 0.06) expressing a significantly higher level of MOR mRNA compared to non-severe patients (0.20 ± 0.03) (*P* = 0.02) (Fig. 1.D).

### The level of lL-17, MCP-1, OPN and IFN-γ in patients

**Fig. 2:**
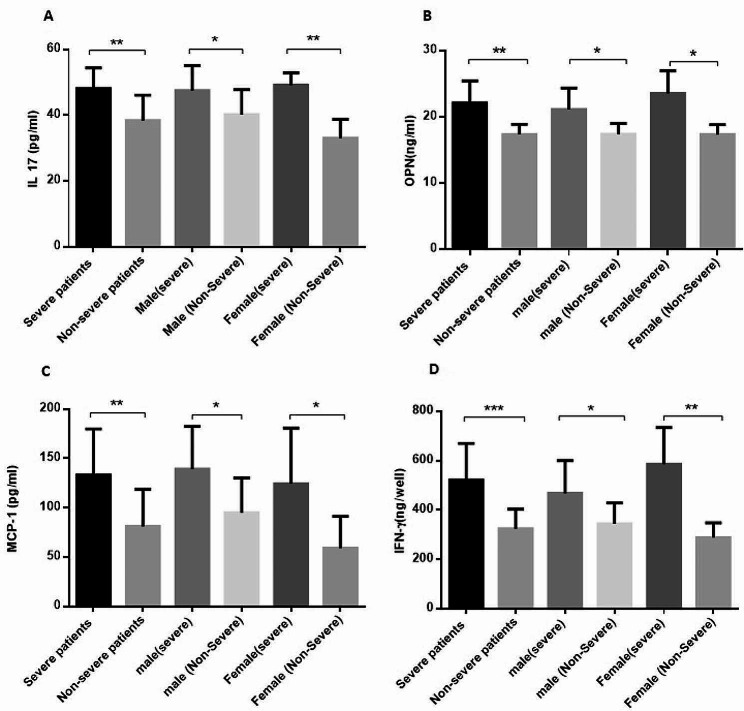
The circulating levels of lL-17, MCP-1, OPN and IFN-γ in patients The levels of lL-17 (Interleukin-17), MCP-1 (Monocyte Chemoattractant Protein-1), OPN (Osteopontin) and IFN-γ (Interferon-γ) were measured in the serum of patients with SARS-CoV-2 (Severe acute respiratory syndrome coronavirus 2) using ELISA assay. The patients were divided into two categories: severe and non-severe; Severe patient had acute clinical manifestations of the disease and high virus concentration in the nasopharyngeal specimen (CT value of gene expression below 20) and were hospitalized; while, non-severe patients had mild respiratory symptoms and low virus concentration in the nasopharyngeal specimen (CT value of gene expression greater than 30) and were not assessed for hospitalization. (**A**): A significant elevated level of lL-17 was detected in severe patients, also in male and female patients with severe infection. (**B**): The OPN level increased in patients with high severity of the disease and in male and female patients with the same pattern, separately. (**C**): An increased level of MCP-1 in patients with severe infection as well as male and female severe patients. (**D**): The level of IFN-γ increased in severe patients also in male and female patients, separately. Statistical significance was determined by *P*-values (two-tailed) and showed by asterisks on bars (*= *P* < 0.05, **= *P* < 0.01, ***= *P* < 0.001)

Our findings demonstrate a significant increase in the circulating level of IL-17 in severe patients (48.10 ± 1.17) compared to non-severe patients (38.39 ± 1.91) (*P* = 0.001) (Fig. 2. A). Similarly, male patients with severe infection (47.47 ± 2.6) exhibited a higher level of circulating IL-17 compared to non-severe male patients (40.18 ± 2.18) (*P* = 0.04), while female patients with severe infection (49.12 ± 1.6) demonstrated a higher level of IL-17 compared to non-severe female patients (33.08 ± 2.85) (*P* = 0.001) (Fig. 2. A). Furthermore, patients with severe SARS-CoV-2 infection had a higher level of OPN (22.11 ± 1.06) compared to non-severe patients (17.37 ± 0.45) (*P* = 0.0005) (Fig. 2.B). When comparing male and female patients, a significant increase in OPN level was observed in male patients with severe infection (21.14 ± 1.31) compared to non-severe counterparts (17.39 ± 0.57) (*P* = 0.014), as well as in female severe patients (23.56 ± 1.72) compared to non-severe female patients (17.33 ± 0.87) (*P* = 0.03) (Fig. 2. B). Additionally, MCP-1 was found to be elevated in patients with severe infection (133.2 ± 14.7) compared to non-severe infection (81 ± 8.8) (*P* = 0.003). This pattern was consistent when comparing male patients with severe infection (139.1 ± 17.73) to non-severe counterparts (94.9 ± 10.66) (*P* = 0.038), and female patients with severe infection (124.5 ± 28.16) to female patients with non-severe infection (59.17 ± 12.17) (*P* = 0.03) (Fig. 2. C). Lastly, the serum level of IFN-γ was significantly enhanced in patients with severe SARS-CoV-2 infection (520.4 ± 33.43) compared to non-severe patients (323.8 ± 21.33) (*P* = 0.0001) (Fig. 2.D). Moreover, an elevated level of IFN-γ was observed in male patients with severe infection (466.9 ± 40.28) compared to male patients with non-severe infection (343.8 ± 28.31) (*P* = 0.02), as well as in female patients with severe infection (585.7 ± 49.50) compared to female patients with non-severe infection (287.8 ± 26.70) (*P* = 0.001) (Fig. 2.D). There was no statistically significant difference in any of the measured variables between male and female patients with severe infection.

### The association of CB2, MOR, lL-17, OPN, MCP-1 and IFN-γ with demographic features of patients and their assessing and predictive values

**Fig. 3:**
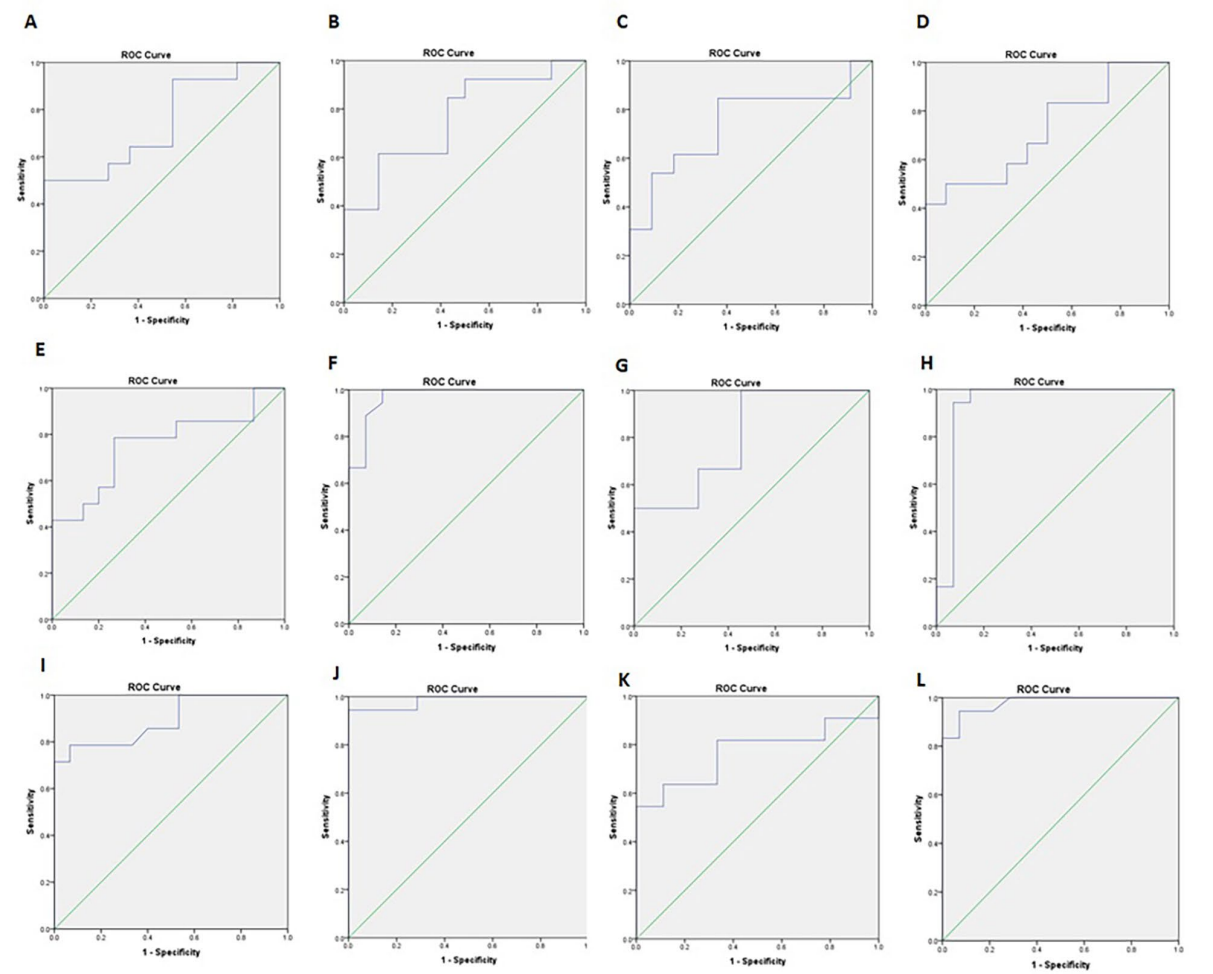
ROC curves of CB2 receptor and MOR gene and lL-17, OPN, MCP-1 and IFN-γ levels between patients The ROC curves of CB2 (Cannabinoid receptor 2) and MOR (μ-opioid receptor) gene levels and lL-17 (Interleukin-17), MCP-1 (Monocyte Chemoattractant Protein-1), OPN (Osteopontin) and IFN-γ (Interferon-γ) levels between different groups are shown. Assessing values were calculated using receiver operating characteristic (ROC) curve analysis, and the area under the curve (AUC) and optimal cut-off values were determined based on the Youden index. Notably, the patients were divided into two categories: severe and non-severe; Severe patient had acute clinical manifestations of the disease and high virus concentration in the nasopharyngeal specimen (CT value of gene expression below 20) and were hospitalized; while, non-severe patients had mild respiratory symptoms and low virus concentration in the nasopharyngeal specimen (CT value of gene expression greater than 30) and were not assessed for hospitalization. (**A**): Indicates CB2 receptor expression level in female group with severe and non-severe SARS-CoV-2 (Severe acute respiratory syndrome coronavirus 2); (**B**): Indicates CB2 receptor expression level in male group with severe and non-severe SARS-CoV-2, (**C**): Indicates MOR expression level in female group with severe and non-severe SARS-CoV-2, (**D**): Indicates MOR expression level in male group with severe and non-severe SARS-CoV-2.(**E**): Indicates lL-17 level in male group with severe and non-severe SARS-CoV-2; (**F**): Indicates lL-17 level in female group with severe and non-severe SARS-CoV-2, (**G**): Indicates MCP-1 level in male group with severe and non-severe SARS-CoV-2, (**H**): Indicates MCP-1 level in female group with severe and non-severe SARS-CoV-2; (I): Indicates OPN level in male group with severe and non-severe SARS-CoV-2; (**J**): Indicates OPN level in female group with severe and non-severe SARS-CoV-2, (**K**): Indicates IFN-γ level in male group with severe and non-severe SARS-CoV-2; (**L**): Indicates IFN-γ level in female group with severe and non-severe infection. Statistical details are presented in Table 2

The assessing values of the measured variables in COVID-19 patients were assessed using ROC curve analysis. The results, including cut-off values, AUC values, sensitivity, specificity, and *P* values, are summarized in Table 2. Figure 3 displays the ROC curves for CB2 and MOR gene levels, and IL-17, OPN, MCP-1, and IFN-γ levels. Our findings indicate that the CB2 receptor level can effectively distinguish between female patients with severe and non-severe infection (*P* = 0.04), as well as between male patients with severe and non-severe infection (*P* = 0.02). Our findings indicate that MOR expression levels can effectively distinguish between male patients with severe and non-severe infections (*P* = 0.04), whereas this distinction was not statistically significant in female patients (*P* = 0.06). Additionally, both male and female patients showed significant variations in IL-17, MCP-1, OPN, and IFN-γ levels, which effectively differentiate between female patients with severe and non-severe infections Furthermore, according to the findings presented in Table 3, the expression level of the CB2 receptor in patients is significantly correlated with the levels of MOR (*P* = 0.0001), MCP-1 (*P* = 0.008), OPN (*P* = 0.042), and IFN-γ (*P* = 0.002). Notably, our data reveals a significant correlation between the MOR expression level in infected patients and the levels of MCP-1 (*P* = 0.011) and IFN-γ (*P* = 0.001). Although no significant correlation was observed between the level of IL-17 and CB2 and MOR, it was significantly associated with the levels of MCP-1, OPN, and IFN-γ (*P* = 0.0001). Based on our data, it appears that the level of MCP-1 is significantly associated with the levels of OPN and IFN-γ (*P* = 0.0001), and there is also a correlation between OPN and IFN-γ (*P* = 0.0001). Thus, the significant correlations between CB2 and MOR receptors and MCP-1, OPN, and IFN-γ suggest the possibility of a cascade effect of these mediators on each other in this infection. Furthermore, a logistic regression model was constructed to assess the predictive value of the independent variables (CB2, MOR, MCP-1, OPN, IL-17, IFN-γ) for determining the severity of SARS-CoV-2 infection (Table 4). Our data demonstrates that the levels of IL-17 (*P* = 0.058), OPN (*P* = 0.003), CB2 (*P* = 0.002), and MOR (*P* = 0.016) can significantly predict disease severity when examined individually and might affect virus replication.

**Table 2:** ROC curve for predicting severity in patients with SARS-CoV-2 via assessing immune relating factors

Variable	Group	Sub-Groups	Cutoff point	Sensitivity (%)	Specificity (%)	AUC	*P*-value	95% CI Lower Bound	95% CI Upper Bound
CB2	Female	Severe Vs. Non-Severe	> 0.151	94	60	0.74	0.04	0.54	0.93
CB2	Male	Severe Vs. Non-Severe	> 0.242	84	62	0.76	0.02	0.58	0.94
MOR	Female	Severe Vs. Non-Severe	> 0.211	66	60	0.72	0.06	0.51	0.92
MOR	Male	Severe Vs. Non-Severe	> 0.295	84	64	0.74	0.04	0.53	0.94
lL-17	Female	Severe Vs. Non-Severe	> 35.44	94	86	0.97	0.0001	0.91	1
lL-17	Male	Severe Vs. Non-Severe	> 42.91	87	67	0.75	0.01	0.57	1
MCP-1	Female	Severe Vs. Non-Severe	> 67.70	100	86	0.93	0.0001	0.82	1
MCP-1	Male	Severe Vs. Non-Severe	> 97.65	83	65	0.80	0.04	0.58	1
OPN	Female	Severe Vs. Non-Severe	> 17.84	94	86	0.98	0.0001	0.94	1
OPN	Male	Severe Vs. Non-Severe	> 18.26	85	60	0.89	0.0001	0.77	1
IFN-γ	Female	Severe Vs. Non-Severe	> 333	94	93	0.93	0.0001	0.93	1
IFN-γ	Male	Severe Vs. Non-Severe	> 350	81	67	0.76	0.04	0.54	0.98

In Table, the Receiver Operating Characteristic (ROC) curve information for CB2, MOR, IL-17, MCP-1, OPN, and IFN-γ in discriminating between severe and non-severe groups of SARS-CoV-2 patients is presented. Key details include:

Cutoff points indicating the threshold values for each biomarker

Sensitivity (%) and Specificity (%) values reflecting the diagnostic accuracy of the biomarkers

The Area Under the Curve (AUC) representing the overall performance of the biomarker in distinguishing between severe and non-severe cases

*P*-values indicating the statistical significance of the AUC values

Confidence interval (CI) displays the probability that a parameter will fall between a pair of values around the mean

These metrics provide valuable insights into the discriminatory power of the biomarkers studied, aiding in the assessment of their predictive potential for SARS-CoV-2 infection severity

**Table 3:** The correlation of CB2, MOR with lL-17, MCP-1, OPN, IFN-γ

		Gene expression		Serum			
	Variable	CB2	MOR	IL-17	MCP-1	OPN	IFN-γ
Gene expression							
	CB2						
	Correlation	1	0.541^**^	0.249	0.335^**^	0.261^*^	0.394^**^
	*P* value		0	0.053	0.008	0.042	0.002
	MOR						
	Correlation	0.541^**^	1	0.206	0.322^*^	0.231	0.424^**^
	*P* value	0		0.112	0.011	0.073	0.001
Serum							
	IL-17						
	Correlation	0.249	0.206	1	0.636^**^	0.476^**^	0.482^**^
	*P* value	0.053	0.112		0	0	0
	MCP-1						
	Correlation	0.335^**^	0.322^*^	0.636^**^	1	0.441^**^	0.646^**^
	*P* value	0.008	0.011	0		0	0
	OPN						
	Correlation	0.261^*^	0.231	0.476^**^	0.441^**^	1	0.636^**^
	*P* value	0.042	0.073	0	0		0
	IFN-γ						
	Correlation	0.394^**^	0.424^**^	0.482^**^	0.646^**^	0.636^**^	1
	*P* value	0.002	0.001	0	0	0	

**. Correlation is significant at the 0.01 level (2-tailed). *. Correlation is significant at the 0.05 level (2-tailed)

Table 3 presents the correlation coefficients and corresponding *P* values for the relationships between CB2, MOR, IL-17, MCP-1, OPN, and IFN-γ. Key points to note include:

Significant correlations (**P < 0.01, *P < 0.05) are denoted by the respective symbols

The correlation values indicate the strength and direction of the relationships between the variables

CB2 and MOR show significant correlations with IL-17, MCP-1, OPN, and IFN-γ, providing insights into their interplay in the context of SARS-CoV-2 infection severity. The analysis method employed for calculating these correlations was Pearson analysis

These correlation results contribute to understanding the potential interactions and associations among the studied biomarkers, shedding light on their roles in the pathophysiology of SARS-CoV-2

**Table 4:** The regression model of variables (Logistic regression)

Dependent variable	Independent variable	OR	95% CI	*P* value
Severity (Severe vs. non-severe)	IL-17	1.208	0.993–1.470	0.058
	MCP-1	0.993	0.953–1.035	0.751
	OPN	2.113	1.280–3.489	0.003
	IFN-γ	1.007	0.995–1.020	0.247
	CB2	124.483	5.700-2718.392	0.002
	MOR	49.264	2.067-1174.294	0.016

Table 4 presents the results of the logistic regression model analyzing the relationship between the dependent variable (Severity - Severe vs. non-severe) and independent variables (IL-17, MCP-1, OPN, IFN-γ, CB2, MOR). Key points to note include:

Odds Ratios (OR) with their corresponding 95% Confidence Intervals (CI) and *P* values are provided for each variable

Logistic regression was utilized to assess the predictive values of the independent variables in determining disease severity

These results contribute to understanding the impact of IL-17, MCP-1, OPN, IFN-γ, CB2, and MOR on the severity of SARS-CoV-2 infection, providing valuable insights into potential prognostic factors

## Discussion

The onset of SARS-CoV-2 infection has been associated with a range of clinical challenges, including disability, hospitalization, high mortality rates, and financial burdens. These issues are partly due to the incomplete comprehension of its intricate pathogenesis and the inadequacy of existing treatment methods [30]. One prevalent theory among many proposed to elucidate the workings of SARS-CoV-2’s pathogenicity highlights the significant involvement of the innate immune system in driving disease advancement, viral replication, and subsequent clinical complexities [31].. Our data showed that The expression of CB2 receptors was higher in both female and male patients with COVID-19, and this was linked to the severity and outcome of the disease. In our current study, we have the opportunity to utilize nasopharynx specimens from patients, which contain epithelial cells that are known to be a major site of virus replication in the respiratory tract. These cells are particularly adapted for acute respiratory infections, and the higher expression of CB2 receptors observed in these cells may suggest their involvement in viral replication and inflammatory conditions [32]. Furthermore, our findings indicated an upregulation of CB2 receptor mRNA in bronchoalveolar lavage (BAL) samples from mice following RSV infection, while there was no change in lung CB2 receptor expression between infected and healthy mice [15].. Interest is growing in the interaction between CB receptors and sex hormones, as well as the shared molecular pathways between estrogen and cannabinoids [33]. Previous studies have indicated that CB2 receptor expression is enhanced in response to 17β-estradiol in osteoclasts, implying a potential direct influence of estrogen on CB receptors [34]. In our study, we observed an increased expression level of CB2 receptors in female patients, which had significant assessing value and showed a correlation with disease outcome. This heightened expression may be related to estrogen levels, though further mechanistic investigations are required to confirm this association. Additionally, we detected an elevated expression of MOR in both male and female patients, which was associated with disease severity. Studies on COVID-19 have shown that opioids interact with the angiotensin-converting enzyme-2 (ACE-2) transmembrane protein, the primary receptor for SARS-CoV-2 entry in host cells. Opioids also interact with Toll-like receptor 4 (TLR4), an innate immune system receptor involved in the immune-pathogenesis of SARS-CoV-2 [35–37]. In our study, we noted an increase in the expression of a critical opioid receptor in patients, which correlated with disease severity. These findings support the potential use of opioid receptor blockers or agonists as treatments for COVID-19, yet further investigation is needed to determine the optimal timing and dosage for such interventions [38, 39].. It is well-established that SARS-CoV-2 infection leads to alterations in the levels of various circulating pro-inflammatory and anti-inflammatory molecules [40].. In line with these findings, our study detected higher levels of MCP-1 in the serum of COVID-19 patients, and this elevation was associated with disease severity. MCP-1 is a well-investigated chemokine that plays a vital role in attracting monocytes and macrophages to inflammatory sites, known to stimulate the expression of inflammatory cytokines [41]. Consistent with our results, a study by Chen Y. et al. also suggested that elevated serum MCP-1 levels in COVID-19 patients may indicate a higher risk of severe disease. It has been proposed that the transcription of the MCP-1 gene is influenced by IFN-γ and the peripheral cannabinoid receptor CB2 [42, 43]. Interestingly, it has been postulated that the transcription of the MCP-1 gene is induced and regulated by IFN-γ [44] as well as the peripheral cannabinoid receptor CB2 [45]. In line with this, our study revealed a significant enhancement of circulating IFN-γ levels in patients with severe infection, which is consistent with the study by Gadotti et al., showing an increase in IFN-γ levels in early stages of severe SARS-CoV-2 infection [46]. Conversely, we observed a significant rise in serum OPN levels in COVID-19 patients, correlating with disease severity. OPN is a glycoprotein that binds to integrin and has multiple functions including cell proliferation, invasion, migration, leucocyte differentiation, and activation. During acute and chronic inflammation, OPN secretion is expected to increase along with other inflammatory cytokines [47, 48]. Activation of the CB2 receptor has been linked to the upregulation of OPN and other mediators in human periodontal ligament cells, suggesting a potential interaction between CB2 receptors and OPN [49]... Additionally, it has been theorized that SARS-CoV-2 infection may directly impact IL-17 through the binding of ORF8 to the IL-17 receptor, triggering IL-17-induced inflammatory responses [50]... Our data consistently showed increased circulating IL-17 levels in patients with severe infection, underscoring the significant role of IL-17 in this disease. Research by Mu et al. indicated that MOR agonist treatment disrupts IL-17-related lymphocytes post-infection, proposing a potential regulatory function of MOR in IL-17-mediated inflammation during infection [51].. Furthermore, the activation of p38 MAPK pathways, leading to MCP-1 production, is a downstream mediator that recruits inflammatory cells and causes lung injury. IL-17 potentially upregulates this process [52].. Additionally, it is suggested that OPN regulates IL-17 production during hepatitis infection, highlighting a regulatory relationship between OPN and IL-17. The concurrent elevation of these compounds in our study’s patients underscores the interconnected regulatory dynamics between OPN and IL-17 [53].. Finally, it should be mentioned that this study had some limitations; first: despite the widespread occurrence of Covid-19, it was necessary for the patients in our study to be individuals who were not undergoing treatment at the time of sampling. Finding such participants was challenging, leading to a meticulous selection process from a large pool of patients, resulting in limited sampling. However, in order to ensure the reliability of the results, it is essential to examine the specified profile in a larger number of patients. Second, the absence of a healthy control group for comparative analysis is the next limitation of this study. While the focus of our research was on assessing the prognostic power of specific molecular markers in predicting the severity of SARS-CoV-2 infection, the inclusion of a healthy control group could have provided valuable insights into the diagnostic potential of the assessed marker. Future studies may benefit from incorporating a healthy control group to enhance the comprehensive evaluation of μ-opioid and cannabinoid receptors, alongside immune mediators, in the context of SARS-CoV-2 infection. Third, it is important to note that crucial clinical markers such as SpO2 and Body Mass Index (BMI) as well as laboratory markers like CRP, D-Dimer, ferritin, IL-6, IL-1B, and TNF-α were not included in the study. These markers could have contributed valuable information regarding the relationship between the assessed markers and established severity biomarkers. Therefore, future research should incorporate these clinical and laboratory markers to improve the thorough assessment of the predictive capabilities of μ-opioid and cannabinoid receptors, in conjunction with immune mediators, in determining the severity of SARS-CoV-2 infection. Fourth, it is recommended to conduct a more comprehensive assessment of the diagnostic and prognostic significance of the factors under investigation in this study. In addition to analyzing ROC, it is important to examine the negative predictive value, as well as evaluate the positive predictive value based on the prevalence of the disease. The fifth, MCP-1, IL-17, IFN-γ, and osteopontin were selected based on their established roles in inflammation, cytokine regulation, and immune response modulation in the context of viral infections. While these markers were chosen for their significance in predicting disease severity in COVID-19, future studies should explore the interplay between other inflammatory mediators associated with cytokine storm to gain a more comprehensive understanding of their impact on SARS-CoV-2 infection. The sixth, further investigation into the gene and protein expression of receptors, including mediator receptors, in relation to SARS-CoV-2 severity across genders is recommended. Additionally, exploring the correlation between receptor expression and clinical indices using robust statistical methods can enhance the depth of future studies in this field.

## Conclusion

The concurrent elevation of OPN, IFN-γ, MCP-1, and IL-17 levels in patients, their association with disease severity, and their assessing significance underscore the importance of these factors. Moreover, the heightened expression of MOR and CB2 receptors indicates potential pharmacological interventions for managing this infection. Further research is necessary to elucidate the involvement of Th1 subtypes and the opioid/cannabinoid systems in preserving immune balance and modulating immune responses in the context of COVID-19.

## Data Availability

All generated data are available within the manuscript and raw data can be provided by Masoumeh Tavakoli-Yaraki upon request.

## Abbreviations

ACE-2: Angiotensin-converting enzyme-2
AUC: Area under the curve
BAL: Bronchoalveolar lavage
CB2: Cannabinoid receptor 2
CB: Cannabinoid receptors
GPCR: G-protein coupled receptor
HRP: Horseradish peroxidase
IFN-γ: Interferon-γ
IFN-γ: Interferon-γ
IL-17: Interleukin-17
MCP-1: Monocyte Chemoattractant Protein-1
MOR: μ-opioid receptor
NK cells: Natural killer cells
OD: Optical density
OPN: Osteopontin
ORF8: Open reading frame 8
PBMC: Peripheral blood monocytes cell
ROC: Receiver operating characteristic curve
RSV: Respiratory syncytial virus
RT-PCR: Real time polymerase chain reaction
SARS-CoV-2: Severe acute respiratory syndrome coronavirus 2
SEM: Standard error mean
TLR4: Toll-like receptor 4
